# (2,2′-Bipyrid­yl-κ^2^ *N*,*N*′)bis­(η^5^-penta­methyl­cyclo­penta­dien­yl)barium

**DOI:** 10.1107/S1600536813017170

**Published:** 2013-07-03

**Authors:** Daniel Kazhdan, Sergio S. Rozenel

**Affiliations:** a Chemistry Department and Chemical Sciences Division of Lawrence Berkeley National Laboratory, University of California, Berkeley, California 94720, USA

## Abstract

In the title compound, [Ba(C_10_H_15_)_2_(C_10_H_8_N_2_)], the Ba—N distances are 2.798 (3) and 2.886 (3) Å, and the Cp ring centroid distances to Ba^2+^ are 2.7291 (7) and 2.7192 (9) Å. The angle between the N atoms in the bypyridine ligand and the metal ion is 56.80 (8)° and the N—C—C—N torsion angle in the bi­pyridine ligand is 1.7 (4)°. The bi­pyridine ligand is almost planar, the dihedral angle formed by the intersection of the planes defined by the pyridyl rings being 3.04 (19)°, and the angle between the plane defined by the Ba^2+^ ion and the two bipyridyl N atoms and the plane defined by the 12 atoms of the bi­pyridine ligand is 10.2 (3)°. The average Ba—N and Ba—centroid distances are 0.16 and 0.14 Å longer, respectively, than the equivalent distances in the isotypic strontium compound [Kazhdan *et al.* (2008 ▶). *Acta Cryst.* E**64**, m1134]. This difference is in accord with the difference between the ionic radii of 0.16 Å suggested by Shannon [*Acta Cryst.* (1976 ▶), A**32**, 751–767].

## Related literature

For the isotypic strontium compound, see: Kazhdan *et al.* (2008 ▶). For ionic radii, see: Shannon (1976 ▶). For the synthesis and related molecules, see: Schultz *et al.* (2002 ▶); Burns & Andersen (1987 ▶).
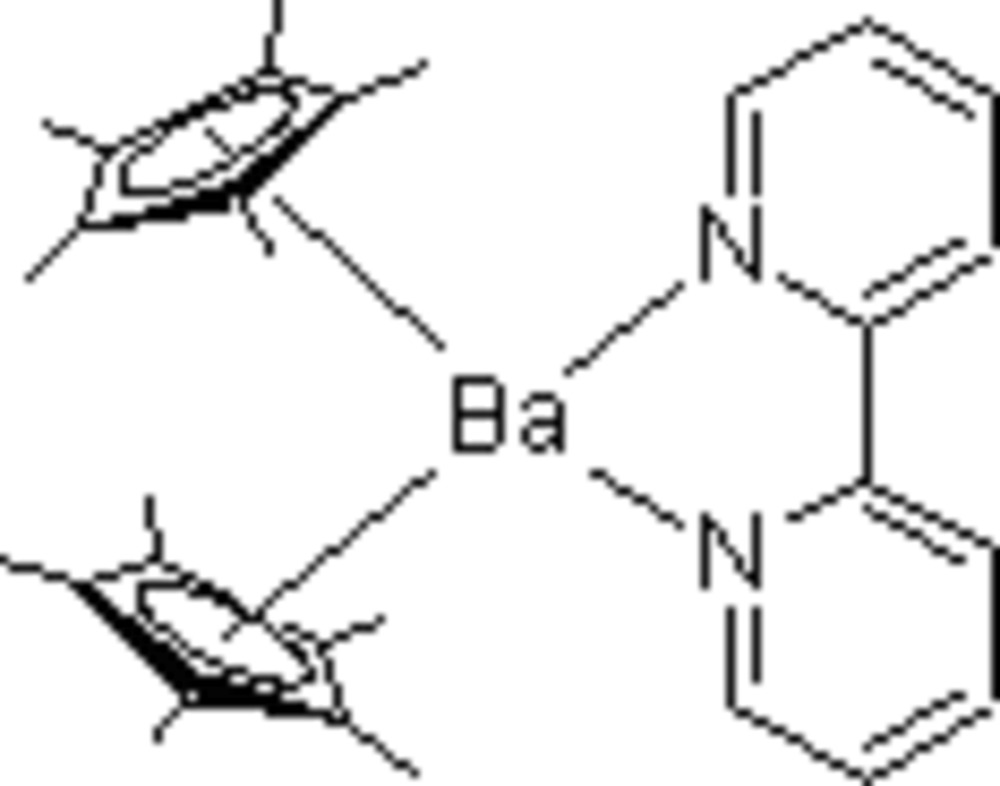


## Experimental

### 

#### Crystal data

[Ba(C_10_H_15_)_2_(C_10_H_8_N_2_)]
*M* *_r_* = 563.96
Orthorhombic, 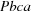

*a* = 15.551 (7) Å
*b* = 17.022 (7) Å
*c* = 20.865 (9) Å
*V* = 5523 (4) Å^3^
*Z* = 8
Mo *K*α radiation
μ = 1.46 mm^−1^
*T* = 173 K
0.18 × 0.15 × 0.12 mm

#### Data collection

Bruker SMART 1000 CCD diffractometer
Absorption correction: multi-scan (*SADABS*; Bruker, 2009 ▶) *T*_min_ = 0.780, *T*_max_ = 0.845
23745 measured reflections
5034 independent reflections
3985 reflections with *I* > 2σ(*I*)
*R*_int_ = 0.031

#### Refinement

*R*[*F* ^2^ > 2σ(*F* ^2^)] = 0.028
*wR*(*F* ^2^) = 0.071
*S* = 1.07
5034 reflections
308 parameters
H-atom parameters constrained
Δρ_max_ = 0.86 e Å^−3^
Δρ_min_ = −0.53 e Å^−3^

### 

Data collection: *APEX2* (Bruker, 2009 ▶); cell refinement: *SAINT* (Bruker, 2009 ▶); data reduction: *SAINT*; program(s) used to solve structure: *SIR97* (Altomare *et al.*, 1999 ▶); program(s) used to refine structure: *SHELXL97* (Sheldrick, 2008 ▶); molecular graphics: *ORTEP-32* (Farrugia, 2012 ▶); software used to prepare material for publication: *WinGX* (Farrugia, 2012 ▶).

## Supplementary Material

Crystal structure: contains datablock(s) global, I. DOI: 10.1107/S1600536813017170/hp2057sup1.cif

Structure factors: contains datablock(s) I. DOI: 10.1107/S1600536813017170/hp2057Isup2.hkl

Additional supplementary materials:  crystallographic information; 3D view; checkCIF report

## Figures and Tables

**Fig. 1.**
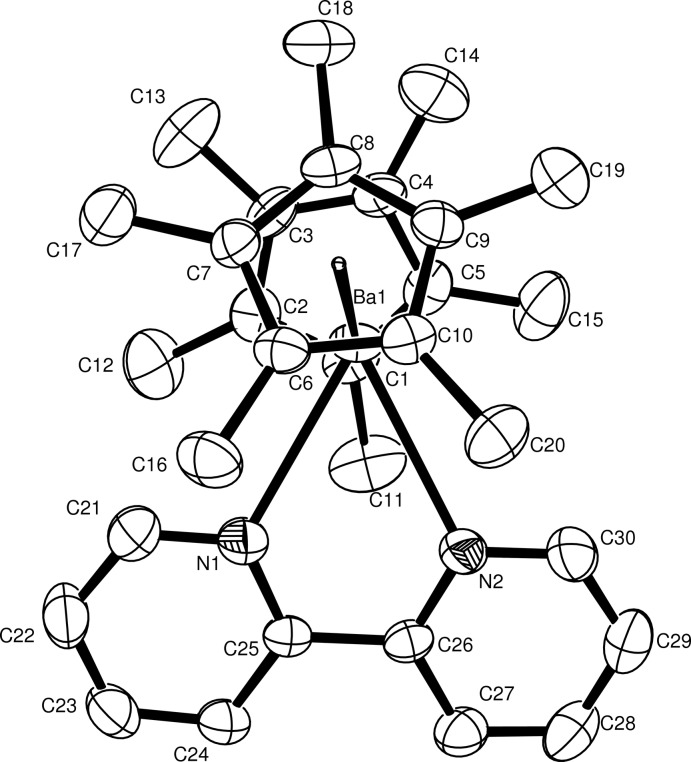
Top view of the structure of (η5-C5Me5)2Ba(bipy), displacement ellipsoids drawn at the 50% probability level. Hydrogen atoms have been removed for clarity.

**Fig. 2.**
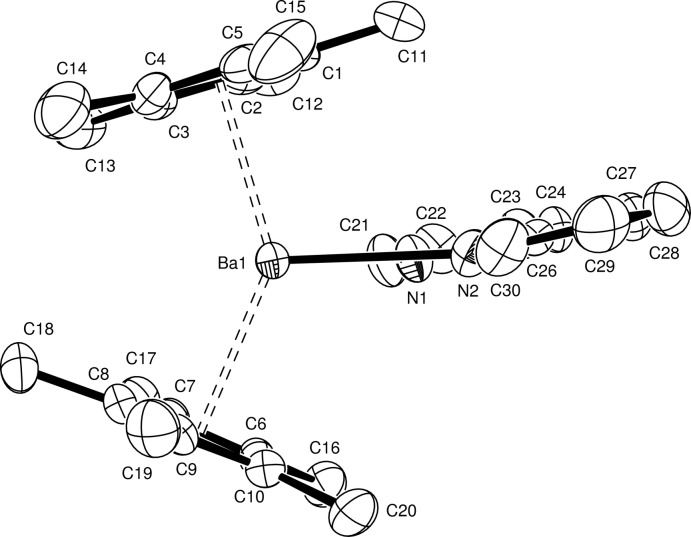
Side view of the structure of (η5-C5Me5)2Ba(bipy), displacement ellipsoids drawn at the 50% probability level. Hydrogen atoms have been removed for clarity.

## supplementary crystallographic information

### Comment

The title compound, [Ba(C10H15)2(C10H8N2)], was crystallized from diethyl ether. The bipyridine ligand is almost planar, and the Ba - Cp ring centroid distances are 2.7276 (6) and 2.7176 (5) Å.

### Experimental

(η^5^-C5Me5)2Ba(bipy) was prepared according to literature procedures (Burns & Andersen, 1987).

### Refinement

All non-hydrogen atoms were refined anisotropically. Hydrogen atoms were fixed based on the expected geometry of the carbon atoms to which they were attached and refined isotropically.

### Crystal data

**Table:**

[Ba(C_10_H_15_)_2_(C_10_H_8_N_2_)]	*F*(000) = 2304
*M**_r_* = 563.96	*D*_x_ = 1.356 Mg m^−^^3^
Orthorhombic, *P**b**c**a*	Mo *K*α radiation, λ = 0.71073 Å
Hall symbol: -P 2ac 2ab	Cell parameters from 5551 reflections
*a* = 15.551 (7) Å	θ = 3.4–26.3°
*b* = 17.022 (7) Å	µ = 1.46 mm^−^^1^
*c* = 20.865 (9) Å	*T* = 173 K
*V* = 5523 (4) Å^3^	Block, red
*Z* = 8	0.18 × 0.15 × 0.12 mm

### Data collection

**Table:**

Bruker SMART 1000 CCD diffractometer	5034 independent reflections
Radiation source: fine-focus sealed tube	3985 reflections with *I* > 2σ(*I*)
Graphite monochromator	*R*_int_ = 0.031
Detector resolution: 8.192 pixels mm^-1^	θ_max_ = 25.5°, θ_min_ = 2.9°
φ and ω scans	*h* = −18→18
Absorption correction: multi-scan (*SADABS*; Bruker, 2009)	*k* = −20→20
*T*_min_ = 0.780, *T*_max_ = 0.845	*l* = −17→24
23745 measured reflections	

### Refinement

**Table:**

Refinement on *F*^2^	Primary atom site location: structure-invariant direct methods
Least-squares matrix: full	Secondary atom site location: difference Fourier map
*R*[*F*^2^ > 2σ(*F*^2^)] = 0.028	Hydrogen site location: inferred from neighbouring sites
*wR*(*F*^2^) = 0.071	H-atom parameters constrained
*S* = 1.07	*w* = 1/[σ^2^(*F*_o_^2^) + (0.0287*P*)^2^ + 6.1469*P*] where *P* = (*F*_o_^2^ + 2*F*_c_^2^)/3
5034 reflections	(Δ/σ)_max_ = 0.010
308 parameters	Δρ_max_ = 0.86 e Å^−^^3^
0 restraints	Δρ_min_ = −0.53 e Å^−^^3^

### Special details

**Table:**

Geometry. All e.s.d.'s (except the e.s.d. in the dihedral angle between two l.s. planes) are estimated using the full covariance matrix. The cell e.s.d.'s are taken into account individually in the estimation of e.s.d.'s in distances, angles and torsion angles; correlations between e.s.d.'s in cell parameters are only used when they are defined by crystal symmetry. An approximate (isotropic) treatment of cell e.s.d.'s is used for estimating e.s.d.'s involving l.s. planes.
Refinement. Refinement of *F*^2^ against ALL reflections. The weighted *R*-factor *wR* and goodness of fit *S* are based on *F*^2^, conventional *R*-factors *R* are based on *F*, with *F* set to zero for negative *F*^2^. The threshold expression of *F*^2^ > σ(*F*^2^) is used only for calculating *R*-factors(gt) *etc*. and is not relevant to the choice of reflections for refinement. *R*-factors based on *F*^2^ are statistically about twice as large as those based on *F*, and *R*- factors based on ALL data will be even larger.

### Fractional atomic coordinates and isotropic or equivalent isotropic displacement parameters (Å^2^)

**Table:**

	*x*	*y*	*z*	*U*_iso_*/*U*_eq_	Occ. (<1)
C1	0.4978 (2)	0.30885 (18)	0.22445 (15)	0.0418 (8)	
C2	0.5648 (2)	0.36266 (18)	0.23698 (15)	0.0417 (8)	
C3	0.5635 (2)	0.42066 (17)	0.18837 (16)	0.0409 (7)	
C4	0.4960 (2)	0.40159 (19)	0.14601 (15)	0.0407 (7)	
C5	0.4554 (2)	0.33260 (19)	0.16825 (16)	0.0419 (8)	
C6	0.78796 (18)	0.24407 (16)	0.04669 (14)	0.0317 (6)	
C7	0.79262 (18)	0.32616 (16)	0.05867 (14)	0.0333 (7)	
C8	0.72765 (19)	0.36359 (16)	0.02191 (14)	0.0326 (6)	
C9	0.68230 (19)	0.30502 (17)	−0.01217 (14)	0.0327 (6)	
C10	0.71974 (19)	0.23139 (15)	0.00309 (14)	0.0311 (6)	
C11	0.4747 (3)	0.2379 (2)	0.26515 (18)	0.0642 (11)	
H11A	0.4251	0.2506	0.2922	0.096*	
H11B	0.5237	0.2238	0.2923	0.096*	
H11C	0.4603	0.1936	0.2372	0.096*	
C12	0.6305 (3)	0.3565 (3)	0.28959 (19)	0.0721 (13)	
H12A	0.6752	0.3190	0.2771	0.108*	
H12B	0.6025	0.3385	0.3290	0.108*	
H12C	0.6565	0.4082	0.2970	0.108*	
C13	0.6215 (3)	0.4914 (2)	0.1844 (2)	0.0668 (11)	
H13A	0.6078	0.5277	0.2193	0.100*	
H13B	0.6128	0.5178	0.1431	0.100*	
H13C	0.6815	0.4746	0.1881	0.100*	
C14	0.4690 (3)	0.4481 (2)	0.08722 (18)	0.0653 (11)	
H14A	0.4891	0.4211	0.0485	0.098*	
H14B	0.4943	0.5007	0.0891	0.098*	
H14C	0.4062	0.4523	0.0861	0.098*	
C15	0.3765 (2)	0.2935 (3)	0.1398 (2)	0.0687 (12)	
H15A	0.3246	0.3175	0.1579	0.103*	
H15B	0.3773	0.2373	0.1500	0.103*	
H15C	0.3767	0.3005	0.0932	0.103*	
C16	0.8454 (2)	0.1815 (2)	0.07456 (17)	0.0470 (8)	
H16A	0.8167	0.1569	0.1113	0.071*	
H16B	0.8996	0.2052	0.0886	0.071*	
H16C	0.8571	0.1415	0.0419	0.071*	
C17	0.8543 (2)	0.3654 (2)	0.10407 (18)	0.0520 (9)	
H17A	0.8380	0.3531	0.1483	0.078*	
H17B	0.8523	0.4224	0.0976	0.078*	
H17C	0.9127	0.3463	0.0959	0.078*	
C18	0.7119 (2)	0.45083 (16)	0.01636 (18)	0.0479 (8)	
H18A	0.7287	0.4688	−0.0265	0.072*	
H18B	0.7461	0.4786	0.0487	0.072*	
H18C	0.6507	0.4618	0.0232	0.072*	
C19	0.6090 (2)	0.3185 (2)	−0.05808 (17)	0.0504 (9)	
H19A	0.5807	0.3683	−0.0478	0.076*	
H19B	0.5675	0.2754	−0.0543	0.076*	
H19C	0.6312	0.3204	−0.1020	0.076*	
C20	0.6929 (2)	0.15298 (18)	−0.02421 (16)	0.0457 (8)	
H20A	0.7320	0.1383	−0.0591	0.069*	
H20B	0.6341	0.1567	−0.0408	0.069*	
H20C	0.6952	0.1130	0.0095	0.069*	
C21	0.7247 (2)	0.14929 (18)	0.25075 (18)	0.0475 (8)	
H21	0.7620	0.1935	0.2506	0.057*	
C22	0.7349 (2)	0.0947 (2)	0.29891 (17)	0.0529 (9)	
H22	0.7768	0.1016	0.3316	0.063*	
C23	0.6819 (2)	0.0298 (2)	0.29790 (17)	0.0540 (9)	
H23	0.6875	−0.0097	0.3298	0.065*	
C24	0.62104 (19)	0.02243 (19)	0.25046 (16)	0.0426 (8)	
H24	0.5843	−0.0221	0.2494	0.051*	
C25	0.61375 (18)	0.08083 (16)	0.20406 (14)	0.0312 (6)	
C26	0.54750 (18)	0.07718 (15)	0.15242 (14)	0.0298 (6)	
C27	0.4904 (2)	0.01508 (18)	0.14691 (16)	0.0420 (8)	
H27	0.4932	−0.0278	0.1760	0.050*	
C28	0.4295 (2)	0.0158 (2)	0.09888 (18)	0.0526 (9)	
H28	0.3904	−0.0267	0.0945	0.063*	
C29	0.4257 (2)	0.0782 (2)	0.05747 (17)	0.0541 (9)	
H29	0.3841	0.0802	0.0242	0.065*	
C30	0.4842 (2)	0.1377 (2)	0.06586 (17)	0.0516 (9)	
H30	0.4819	0.1811	0.0373	0.062*	
CT01	0.7420	0.2940	0.0236	0.010*	0.00
CT02	0.5155	0.3653	0.1928	0.010*	0.00
N1	0.66595 (16)	0.14438 (13)	0.20414 (13)	0.0372 (6)	
N2	0.54426 (17)	0.13841 (14)	0.11168 (12)	0.0404 (6)	
Ba1	0.633367 (11)	0.280277 (9)	0.125109 (8)	0.03061 (7)	

### Atomic displacement parameters (Å^2^)

**Table:**

	*U*^11^	*U*^22^	*U*^33^	*U*^12^	*U*^13^	*U*^23^
C1	0.047 (2)	0.0410 (17)	0.0373 (18)	0.0105 (15)	0.0158 (15)	0.0015 (14)
C2	0.051 (2)	0.0445 (17)	0.0300 (17)	0.0157 (15)	−0.0014 (14)	−0.0079 (14)
C3	0.0455 (19)	0.0339 (16)	0.0433 (19)	0.0075 (13)	0.0039 (15)	−0.0039 (13)
C4	0.0402 (19)	0.0460 (18)	0.0359 (17)	0.0180 (14)	0.0048 (14)	0.0030 (14)
C5	0.0315 (17)	0.0503 (19)	0.0438 (19)	0.0073 (14)	0.0071 (14)	−0.0022 (15)
C6	0.0295 (16)	0.0305 (14)	0.0351 (17)	0.0027 (11)	0.0058 (13)	0.0011 (12)
C7	0.0294 (16)	0.0341 (16)	0.0363 (16)	−0.0059 (12)	0.0038 (13)	0.0040 (12)
C8	0.0343 (16)	0.0278 (14)	0.0355 (16)	−0.0017 (12)	0.0065 (13)	0.0078 (12)
C9	0.0337 (17)	0.0328 (15)	0.0314 (16)	−0.0006 (12)	0.0001 (13)	0.0067 (12)
C10	0.0369 (17)	0.0281 (15)	0.0282 (15)	−0.0020 (12)	0.0046 (13)	−0.0021 (11)
C11	0.082 (3)	0.057 (2)	0.054 (2)	0.013 (2)	0.036 (2)	0.0120 (18)
C12	0.091 (3)	0.075 (3)	0.051 (2)	0.034 (2)	−0.027 (2)	−0.023 (2)
C13	0.073 (3)	0.045 (2)	0.083 (3)	−0.0079 (18)	0.006 (2)	−0.016 (2)
C14	0.072 (3)	0.075 (3)	0.049 (2)	0.034 (2)	0.005 (2)	0.017 (2)
C15	0.032 (2)	0.088 (3)	0.086 (3)	−0.0001 (18)	0.0002 (19)	−0.008 (2)
C16	0.044 (2)	0.0462 (19)	0.051 (2)	0.0145 (15)	0.0002 (16)	0.0049 (16)
C17	0.049 (2)	0.053 (2)	0.054 (2)	−0.0131 (16)	−0.0090 (17)	−0.0005 (17)
C18	0.051 (2)	0.0289 (16)	0.064 (2)	−0.0023 (14)	0.0065 (18)	0.0097 (15)
C19	0.052 (2)	0.053 (2)	0.047 (2)	0.0005 (16)	−0.0112 (17)	0.0110 (16)
C20	0.059 (2)	0.0347 (17)	0.0432 (19)	−0.0018 (15)	0.0013 (16)	−0.0104 (14)
C21	0.0352 (18)	0.0417 (18)	0.066 (2)	−0.0017 (14)	−0.0103 (17)	−0.0020 (16)
C22	0.040 (2)	0.067 (2)	0.052 (2)	0.0073 (17)	−0.0116 (17)	0.0016 (18)
C23	0.049 (2)	0.062 (2)	0.051 (2)	0.0083 (18)	0.0001 (18)	0.0205 (18)
C24	0.0371 (18)	0.0413 (18)	0.049 (2)	−0.0010 (13)	0.0009 (15)	0.0110 (15)
C25	0.0272 (15)	0.0309 (15)	0.0355 (16)	0.0026 (11)	0.0062 (12)	0.0004 (12)
C26	0.0285 (15)	0.0265 (14)	0.0344 (16)	0.0010 (11)	0.0050 (12)	−0.0004 (12)
C27	0.045 (2)	0.0350 (17)	0.0456 (19)	−0.0072 (14)	−0.0021 (15)	0.0032 (14)
C28	0.053 (2)	0.049 (2)	0.055 (2)	−0.0185 (16)	−0.0082 (18)	−0.0024 (17)
C29	0.050 (2)	0.068 (2)	0.044 (2)	−0.0118 (18)	−0.0131 (17)	0.0016 (17)
C30	0.059 (2)	0.049 (2)	0.046 (2)	−0.0084 (17)	−0.0114 (18)	0.0123 (16)
N1	0.0302 (14)	0.0326 (13)	0.0487 (16)	−0.0001 (10)	−0.0022 (12)	0.0021 (11)
N2	0.0420 (16)	0.0378 (14)	0.0413 (16)	−0.0078 (11)	−0.0026 (12)	0.0075 (11)
Ba1	0.03427 (11)	0.02186 (10)	0.03571 (11)	−0.00059 (6)	0.00936 (7)	0.00253 (7)

### Geometric parameters (Å, º)

**Table:**

C1—CT02	1.197 (3)	C13—H13C	0.9800
C1—C5	1.404 (4)	C14—H14A	0.9800
C1—C2	1.412 (5)	C14—H14B	0.9800
C1—C11	1.519 (4)	C14—H14C	0.9800
C1—Ba1	2.996 (3)	C15—H15A	0.9800
C2—CT02	1.200 (3)	C15—H15B	0.9800
C2—C3	1.416 (4)	C15—H15C	0.9800
C2—C12	1.502 (5)	C16—H16A	0.9800
C2—Ba1	2.924 (3)	C16—H16B	0.9800
C3—CT02	1.206 (3)	C16—H16C	0.9800
C3—C4	1.409 (5)	C17—H17A	0.9800
C3—C13	1.506 (5)	C17—H17B	0.9800
C3—Ba1	2.938 (3)	C17—H17C	0.9800
C4—CT02	1.195 (3)	C18—H18A	0.9800
C4—C5	1.412 (5)	C18—H18B	0.9800
C4—C14	1.519 (4)	C18—H18C	0.9800
C4—Ba1	3.003 (3)	C19—H19A	0.9800
C5—CT02	1.202 (3)	C19—H19B	0.9800
C5—C15	1.517 (5)	C19—H19C	0.9800
C5—Ba1	3.043 (3)	C20—H20A	0.9800
C6—CT01	1.210 (3)	C20—H20B	0.9800
C6—C10	1.414 (4)	C20—H20C	0.9800
C6—C7	1.421 (4)	C21—N1	1.337 (4)
C6—C16	1.507 (4)	C21—C22	1.377 (5)
C6—Ba1	2.973 (3)	C21—H21	0.9500
C7—CT01	1.205 (3)	C22—C23	1.379 (5)
C7—C8	1.419 (4)	C22—H22	0.9500
C7—C17	1.504 (4)	C23—C24	1.375 (5)
C7—Ba1	2.944 (3)	C23—H23	0.9500
C8—CT01	1.205 (3)	C24—C25	1.392 (4)
C8—C9	1.413 (4)	C24—H24	0.9500
C8—C18	1.509 (4)	C25—N1	1.353 (4)
C8—Ba1	2.966 (3)	C25—C26	1.492 (4)
C9—CT01	1.207 (3)	C26—N2	1.346 (4)
C9—C10	1.418 (4)	C26—C27	1.385 (4)
C9—C19	1.506 (4)	C27—C28	1.379 (5)
C9—Ba1	2.994 (3)	C27—H27	0.9500
C10—CT01	1.201 (3)	C28—C29	1.370 (5)
C10—C20	1.510 (4)	C28—H28	0.9500
C10—Ba1	2.996 (3)	C29—C30	1.373 (5)
C11—H11A	0.9800	C29—H29	0.9500
C11—H11B	0.9800	C30—N2	1.336 (4)
C11—H11C	0.9800	C30—H30	0.9500
C12—H12A	0.9800	CT01—Ba1	2.7192 (9)
C12—H12B	0.9800	CT02—Ba1	2.7291 (7)
C12—H12C	0.9800	N1—Ba1	2.886 (3)
C13—H13A	0.9800	N2—Ba1	2.798 (3)
C13—H13B	0.9800		
			
CT02—C1—C5	54.32 (19)	H18A—C18—H18C	109.5
CT02—C1—C2	54.00 (19)	H18B—C18—H18C	109.5
C5—C1—C2	108.3 (3)	C9—C19—H19A	109.5
CT02—C1—C11	179.3 (3)	C9—C19—H19B	109.5
C5—C1—C11	125.7 (3)	H19A—C19—H19B	109.5
C2—C1—C11	125.9 (3)	C9—C19—H19C	109.5
CT02—C1—Ba1	65.62 (13)	H19A—C19—H19C	109.5
C5—C1—Ba1	78.42 (17)	H19B—C19—H19C	109.5
C2—C1—Ba1	73.38 (17)	C10—C20—H20A	109.5
C11—C1—Ba1	115.1 (2)	C10—C20—H20B	109.5
CT02—C2—C1	53.84 (19)	H20A—C20—H20B	109.5
CT02—C2—C3	54.13 (18)	C10—C20—H20C	109.5
C1—C2—C3	108.0 (3)	H20A—C20—H20C	109.5
CT02—C2—C12	176.4 (3)	H20B—C20—H20C	109.5
C1—C2—C12	126.3 (3)	N1—C21—C22	124.6 (3)
C3—C2—C12	125.6 (3)	N1—C21—H21	117.7
CT02—C2—Ba1	68.76 (13)	C22—C21—H21	117.7
C1—C2—Ba1	79.06 (17)	C21—C22—C23	117.4 (3)
C3—C2—Ba1	76.59 (17)	C21—C22—H22	121.3
C12—C2—Ba1	107.6 (2)	C23—C22—H22	121.3
CT02—C3—C4	53.69 (19)	C24—C23—C22	119.7 (3)
CT02—C3—C2	53.76 (19)	C24—C23—H23	120.2
C4—C3—C2	107.5 (3)	C22—C23—H23	120.2
CT02—C3—C13	178.1 (3)	C23—C24—C25	119.4 (3)
C4—C3—C13	126.5 (3)	C23—C24—H24	120.3
C2—C3—C13	126.0 (3)	C25—C24—H24	120.3
CT02—C3—Ba1	68.13 (13)	N1—C25—C24	121.4 (3)
C4—C3—Ba1	78.84 (17)	N1—C25—C26	116.7 (2)
C2—C3—Ba1	75.46 (17)	C24—C25—C26	121.9 (3)
C13—C3—Ba1	113.8 (2)	N2—C26—C27	121.0 (3)
CT02—C4—C3	54.40 (18)	N2—C26—C25	116.7 (2)
CT02—C4—C5	54.15 (19)	C27—C26—C25	122.3 (3)
C3—C4—C5	108.5 (3)	C28—C27—C26	119.6 (3)
CT02—C4—C14	178.6 (3)	C28—C27—H27	120.2
C3—C4—C14	126.3 (3)	C26—C27—H27	120.2
C5—C4—C14	125.1 (3)	C29—C28—C27	119.7 (3)
CT02—C4—Ba1	65.31 (13)	C29—C28—H28	120.1
C3—C4—Ba1	73.74 (17)	C27—C28—H28	120.1
C5—C4—Ba1	78.08 (17)	C28—C29—C30	117.5 (3)
C14—C4—Ba1	116.0 (2)	C28—C29—H29	121.2
CT02—C5—C1	54.03 (19)	C30—C29—H29	121.2
CT02—C5—C4	53.69 (19)	N2—C30—C29	124.1 (3)
C1—C5—C4	107.7 (3)	N2—C30—H30	117.9
CT02—C5—C15	176.9 (3)	C29—C30—H30	117.9
C1—C5—C15	125.4 (3)	C10—CT01—C7	143.9 (2)
C4—C5—C15	126.7 (3)	C10—CT01—C8	143.9 (2)
CT02—C5—Ba1	63.57 (13)	C7—CT01—C8	72.17 (19)
C1—C5—Ba1	74.70 (17)	C10—CT01—C9	72.19 (19)
C4—C5—Ba1	74.93 (17)	C7—CT01—C9	143.89 (19)
C15—C5—Ba1	119.4 (2)	C8—CT01—C9	71.73 (19)
CT01—C6—C10	53.77 (16)	C10—CT01—C6	71.8 (2)
CT01—C6—C7	53.77 (17)	C7—CT01—C6	72.1 (2)
C10—C6—C7	107.5 (2)	C8—CT01—C6	144.3 (2)
CT01—C6—C16	179.3 (3)	C9—CT01—C6	144.00 (19)
C10—C6—C16	125.8 (3)	C10—CT01—Ba1	91.25 (14)
C7—C6—C16	126.7 (3)	C7—CT01—Ba1	88.42 (14)
CT01—C6—Ba1	66.17 (12)	C8—CT01—Ba1	89.57 (13)
C10—C6—Ba1	77.23 (17)	C9—CT01—Ba1	90.97 (14)
C7—C6—Ba1	74.97 (16)	C6—CT01—Ba1	89.80 (14)
C16—C6—Ba1	114.4 (2)	C4—CT02—C1	143.8 (2)
CT01—C7—C8	53.93 (17)	C4—CT02—C2	144.0 (2)
CT01—C7—C6	54.13 (16)	C1—CT02—C2	72.2 (2)
C8—C7—C6	108.1 (2)	C4—CT02—C5	72.2 (2)
CT01—C7—C17	178.3 (3)	C1—CT02—C5	71.7 (2)
C8—C7—C17	126.5 (3)	C2—CT02—C5	143.8 (2)
C6—C7—C17	125.4 (3)	C4—CT02—C3	71.9 (2)
CT01—C7—Ba1	67.43 (12)	C1—CT02—C3	144.3 (2)
C8—C7—Ba1	76.98 (16)	C2—CT02—C3	72.1 (2)
C6—C7—Ba1	77.24 (16)	C5—CT02—C3	144.1 (2)
C17—C7—Ba1	111.0 (2)	C4—CT02—Ba1	91.25 (14)
CT01—C8—C9	54.18 (16)	C1—CT02—Ba1	90.83 (14)
CT01—C8—C7	53.90 (16)	C2—CT02—Ba1	87.05 (14)
C9—C8—C7	108.1 (2)	C5—CT02—Ba1	93.21 (14)
CT01—C8—C18	177.0 (3)	C3—CT02—Ba1	87.67 (15)
C9—C8—C18	125.1 (3)	C21—N1—C25	117.5 (3)
C7—C8—C18	126.8 (3)	C21—N1—Ba1	119.04 (19)
CT01—C8—Ba1	66.46 (12)	C25—N1—Ba1	122.35 (19)
C9—C8—Ba1	77.37 (16)	C30—N2—C26	118.1 (3)
C7—C8—Ba1	75.23 (16)	C30—N2—Ba1	115.2 (2)
C18—C8—Ba1	116.5 (2)	C26—N2—Ba1	125.91 (19)
CT01—C9—C8	54.09 (16)	CT01—Ba1—CT02	140.781 (16)
CT01—C9—C10	53.71 (16)	CT01—Ba1—N2	107.71 (6)
C8—C9—C10	107.8 (3)	CT02—Ba1—N2	100.18 (6)
CT01—C9—C19	178.8 (3)	CT01—Ba1—N1	113.88 (5)
C8—C9—C19	126.2 (3)	CT02—Ba1—N1	104.31 (6)
C10—C9—C19	126.0 (3)	N2—Ba1—N1	56.80 (8)
CT01—C9—Ba1	65.26 (12)	CT01—Ba1—C2	143.78 (7)
C8—C9—Ba1	75.20 (16)	CT02—Ba1—C2	24.19 (6)
C10—C9—Ba1	76.42 (16)	N2—Ba1—C2	108.26 (9)
C19—C9—Ba1	116.0 (2)	N1—Ba1—C2	89.56 (8)
CT01—C10—C6	54.41 (16)	CT01—Ba1—C3	120.64 (7)
CT01—C10—C9	54.11 (16)	CT02—Ba1—C3	24.20 (6)
C6—C10—C9	108.5 (2)	N2—Ba1—C3	124.30 (9)
CT01—C10—C20	178.6 (3)	N1—Ba1—C3	117.40 (9)
C6—C10—C20	125.8 (3)	C2—Ba1—C3	27.95 (8)
C9—C10—C20	125.7 (3)	CT01—Ba1—C7	24.15 (6)
CT01—C10—Ba1	65.13 (12)	CT02—Ba1—C7	131.92 (6)
C6—C10—Ba1	75.36 (16)	N2—Ba1—C7	126.76 (8)
C9—C10—Ba1	76.19 (16)	N1—Ba1—C7	109.51 (8)
C20—C10—Ba1	116.24 (19)	C2—Ba1—C7	123.73 (9)
C1—C11—H11A	109.5	C3—Ba1—C7	107.88 (9)
C1—C11—H11B	109.5	CT01—Ba1—C8	23.98 (5)
H11A—C11—H11B	109.5	CT02—Ba1—C8	117.02 (6)
C1—C11—H11C	109.5	N2—Ba1—C8	125.78 (8)
H11A—C11—H11C	109.5	N1—Ba1—C8	135.34 (8)
H11B—C11—H11C	109.5	C2—Ba1—C8	122.03 (9)
C2—C12—H12A	109.5	C3—Ba1—C8	96.90 (9)
C2—C12—H12B	109.5	C7—Ba1—C8	27.79 (8)
H12A—C12—H12B	109.5	CT01—Ba1—C6	24.03 (6)
C2—C12—H12C	109.5	CT02—Ba1—C6	159.71 (6)
H12A—C12—H12C	109.5	N2—Ba1—C6	99.58 (8)
H12B—C12—H12C	109.5	N1—Ba1—C6	90.36 (8)
C3—C13—H13A	109.5	C2—Ba1—C6	146.46 (9)
C3—C13—H13B	109.5	C3—Ba1—C6	135.65 (9)
H13A—C13—H13B	109.5	C7—Ba1—C6	27.80 (8)
C3—C13—H13C	109.5	C8—Ba1—C6	45.56 (8)
H13A—C13—H13C	109.5	CT01—Ba1—C9	23.77 (6)
H13B—C13—H13C	109.5	CT02—Ba1—C9	126.26 (6)
C4—C14—H14A	109.5	N2—Ba1—C9	98.71 (8)
C4—C14—H14B	109.5	N1—Ba1—C9	127.95 (8)
H14A—C14—H14B	109.5	C2—Ba1—C9	142.09 (8)
C4—C14—H14C	109.5	C3—Ba1—C9	114.17 (9)
H14A—C14—H14C	109.5	C7—Ba1—C9	45.43 (8)
H14B—C14—H14C	109.5	C8—Ba1—C9	27.43 (8)
C5—C15—H15A	109.5	C6—Ba1—C9	45.33 (8)
C5—C15—H15B	109.5	CT01—Ba1—C1	164.15 (6)
H15A—C15—H15B	109.5	CT02—Ba1—C1	23.56 (6)
C5—C15—H15C	109.5	N2—Ba1—C1	82.01 (9)
H15A—C15—H15C	109.5	N1—Ba1—C1	81.86 (8)
H15B—C15—H15C	109.5	C2—Ba1—C1	27.56 (9)
C6—C16—H16A	109.5	C3—Ba1—C1	45.32 (9)
C6—C16—H16B	109.5	C7—Ba1—C1	151.01 (9)
H16A—C16—H16B	109.5	C8—Ba1—C1	140.57 (8)
C6—C16—H16C	109.5	C6—Ba1—C1	169.57 (9)
H16A—C16—H16C	109.5	C9—Ba1—C1	144.88 (8)
H16B—C16—H16C	109.5	CT01—Ba1—C10	23.62 (5)
C7—C17—H17A	109.5	CT02—Ba1—C10	152.63 (6)
C7—C17—H17B	109.5	N2—Ba1—C10	84.10 (8)
H17A—C17—H17B	109.5	N1—Ba1—C10	100.61 (8)
C7—C17—H17C	109.5	C2—Ba1—C10	167.11 (8)
H17A—C17—H17C	109.5	C3—Ba1—C10	140.66 (8)
H17B—C17—H17C	109.5	C7—Ba1—C10	45.28 (8)
C8—C18—H18A	109.5	C8—Ba1—C10	45.12 (8)
C8—C18—H18B	109.5	C6—Ba1—C10	27.40 (8)
H18A—C18—H18B	109.5	C9—Ba1—C10	27.39 (8)
C8—C18—H18C	109.5	C1—Ba1—C10	161.50 (9)
			
C5—C1—C2—CT02	0.2 (2)	C1—CT02—Ba1—C3	−144.3 (2)
C11—C1—C2—CT02	−179.1 (3)	C2—CT02—Ba1—C3	−72.2 (2)
Ba1—C1—C2—CT02	71.60 (12)	C5—CT02—Ba1—C3	144.0 (2)
CT02—C1—C2—C3	0.3 (2)	C4—CT02—Ba1—C7	64.25 (19)
C5—C1—C2—C3	0.5 (3)	C1—CT02—Ba1—C7	−151.87 (19)
C11—C1—C2—C3	−178.8 (3)	C2—CT02—Ba1—C7	−79.77 (18)
Ba1—C1—C2—C3	71.9 (2)	C5—CT02—Ba1—C7	136.46 (18)
CT02—C1—C2—C12	−175.5 (4)	C3—CT02—Ba1—C7	−7.58 (17)
C5—C1—C2—C12	−175.3 (3)	C4—CT02—Ba1—C8	35.50 (18)
C11—C1—C2—C12	5.4 (5)	C1—CT02—Ba1—C8	179.38 (18)
Ba1—C1—C2—C12	−103.9 (3)	C2—CT02—Ba1—C8	−108.53 (17)
CT02—C1—C2—Ba1	−71.60 (12)	C5—CT02—Ba1—C8	107.71 (17)
C5—C1—C2—Ba1	−71.4 (2)	C3—CT02—Ba1—C8	−36.33 (17)
C11—C1—C2—Ba1	109.3 (3)	C4—CT02—Ba1—C6	63.0 (2)
C1—C2—C3—CT02	−0.28 (19)	C1—CT02—Ba1—C6	−153.2 (2)
C12—C2—C3—CT02	175.5 (4)	C2—CT02—Ba1—C6	−81.1 (2)
Ba1—C2—C3—CT02	73.31 (12)	C5—CT02—Ba1—C6	135.2 (2)
CT02—C2—C3—C4	−0.24 (19)	C3—CT02—Ba1—C6	−8.9 (2)
C1—C2—C3—C4	−0.5 (3)	C4—CT02—Ba1—C9	5.00 (18)
C12—C2—C3—C4	175.3 (3)	C1—CT02—Ba1—C9	148.87 (18)
Ba1—C2—C3—C4	73.1 (2)	C2—CT02—Ba1—C9	−139.03 (17)
CT02—C2—C3—C13	177.6 (4)	C5—CT02—Ba1—C9	77.20 (17)
C1—C2—C3—C13	177.3 (3)	C3—CT02—Ba1—C9	−66.84 (17)
C12—C2—C3—C13	−6.8 (5)	C4—CT02—Ba1—C1	−143.9 (2)
Ba1—C2—C3—C13	−109.1 (3)	C2—CT02—Ba1—C1	72.1 (2)
CT02—C2—C3—Ba1	−73.31 (12)	C5—CT02—Ba1—C1	−71.7 (2)
C1—C2—C3—Ba1	−73.6 (2)	C3—CT02—Ba1—C1	144.3 (2)
C12—C2—C3—Ba1	102.2 (3)	C4—CT02—Ba1—C10	−6.9 (2)
C2—C3—C4—CT02	0.24 (19)	C1—CT02—Ba1—C10	136.9 (2)
C13—C3—C4—CT02	−177.6 (4)	C2—CT02—Ba1—C10	−151.0 (2)
Ba1—C3—C4—CT02	70.96 (12)	C5—CT02—Ba1—C10	65.3 (2)
CT02—C3—C4—C5	0.1 (2)	C3—CT02—Ba1—C10	−78.8 (2)
C2—C3—C4—C5	0.3 (3)	C30—N2—Ba1—CT01	−73.0 (2)
C13—C3—C4—C5	−177.5 (3)	C26—N2—Ba1—CT01	117.5 (2)
Ba1—C3—C4—C5	71.1 (2)	C30—N2—Ba1—CT02	79.0 (2)
CT02—C3—C4—C14	178.5 (4)	C26—N2—Ba1—CT02	−90.4 (2)
C2—C3—C4—C14	178.7 (3)	C30—N2—Ba1—N1	179.6 (3)
C13—C3—C4—C14	0.9 (5)	C26—N2—Ba1—N1	10.2 (2)
Ba1—C3—C4—C14	−110.6 (3)	C30—N2—Ba1—C2	102.6 (2)
CT02—C3—C4—Ba1	−70.96 (12)	C26—N2—Ba1—C2	−66.8 (2)
C2—C3—C4—Ba1	−70.7 (2)	C30—N2—Ba1—C3	76.9 (3)
C13—C3—C4—Ba1	111.4 (3)	C26—N2—Ba1—C3	−92.5 (2)
C2—C1—C5—CT02	−0.23 (19)	C30—N2—Ba1—C7	−89.9 (3)
C11—C1—C5—CT02	179.1 (3)	C26—N2—Ba1—C7	100.7 (2)
Ba1—C1—C5—CT02	−68.18 (12)	C30—N2—Ba1—C8	−55.3 (3)
CT02—C1—C5—C4	−0.07 (19)	C26—N2—Ba1—C8	135.3 (2)
C2—C1—C5—C4	−0.3 (3)	C30—N2—Ba1—C6	−96.3 (2)
C11—C1—C5—C4	179.0 (3)	C26—N2—Ba1—C6	94.2 (2)
Ba1—C1—C5—C4	−68.3 (2)	C30—N2—Ba1—C9	−50.4 (2)
CT02—C1—C5—C15	−176.3 (4)	C26—N2—Ba1—C9	140.2 (2)
C2—C1—C5—C15	−176.5 (3)	C30—N2—Ba1—C1	94.1 (2)
C11—C1—C5—C15	2.8 (5)	C26—N2—Ba1—C1	−75.3 (2)
Ba1—C1—C5—C15	115.5 (3)	C30—N2—Ba1—C10	−73.6 (2)
CT02—C1—C5—Ba1	68.18 (12)	C26—N2—Ba1—C10	116.9 (2)
C2—C1—C5—Ba1	68.0 (2)	C21—N1—Ba1—CT01	85.7 (2)
C11—C1—C5—Ba1	−112.7 (3)	C25—N1—Ba1—CT01	−106.9 (2)
C3—C4—C5—CT02	−0.1 (2)	C21—N1—Ba1—CT02	−85.2 (2)
C14—C4—C5—CT02	−178.5 (4)	C25—N1—Ba1—CT02	82.2 (2)
Ba1—C4—C5—CT02	68.03 (12)	C21—N1—Ba1—N2	−178.1 (3)
CT02—C4—C5—C1	0.07 (19)	C25—N1—Ba1—N2	−10.7 (2)
C3—C4—C5—C1	0.0 (3)	C21—N1—Ba1—C2	−65.8 (2)
C14—C4—C5—C1	−178.4 (3)	C25—N1—Ba1—C2	101.6 (2)
Ba1—C4—C5—C1	68.1 (2)	C21—N1—Ba1—C3	−63.3 (3)
CT02—C4—C5—C15	176.2 (4)	C25—N1—Ba1—C3	104.1 (2)
C3—C4—C5—C15	176.1 (3)	C21—N1—Ba1—C7	60.1 (2)
C14—C4—C5—C15	−2.3 (5)	C25—N1—Ba1—C7	−132.5 (2)
Ba1—C4—C5—C15	−115.7 (3)	C21—N1—Ba1—C8	72.5 (3)
CT02—C4—C5—Ba1	−68.03 (12)	C25—N1—Ba1—C8	−120.1 (2)
C3—C4—C5—Ba1	−68.1 (2)	C21—N1—Ba1—C6	80.6 (2)
C14—C4—C5—Ba1	113.5 (3)	C25—N1—Ba1—C6	−111.9 (2)
C10—C6—C7—CT01	−0.18 (18)	C21—N1—Ba1—C9	108.2 (2)
C16—C6—C7—CT01	179.3 (3)	C25—N1—Ba1—C9	−84.4 (2)
Ba1—C6—C7—CT01	−71.22 (12)	C21—N1—Ba1—C1	−92.4 (2)
CT01—C6—C7—C8	−0.30 (18)	C25—N1—Ba1—C1	75.0 (2)
C10—C6—C7—C8	−0.5 (3)	C21—N1—Ba1—C10	106.2 (2)
C16—C6—C7—C8	179.0 (3)	C25—N1—Ba1—C10	−86.4 (2)
Ba1—C6—C7—C8	−71.5 (2)	CT02—C2—Ba1—CT01	99.23 (17)
CT01—C6—C7—C17	178.0 (3)	C1—C2—Ba1—CT01	154.51 (14)
C10—C6—C7—C17	177.8 (3)	C3—C2—Ba1—CT01	42.8 (2)
C16—C6—C7—C17	−2.7 (5)	C12—C2—Ba1—CT01	−80.7 (3)
Ba1—C6—C7—C17	106.8 (3)	C1—C2—Ba1—CT02	55.27 (19)
CT01—C6—C7—Ba1	71.22 (12)	C3—C2—Ba1—CT02	−56.39 (18)
C10—C6—C7—Ba1	71.0 (2)	C12—C2—Ba1—CT02	−179.9 (4)
C16—C6—C7—Ba1	−109.5 (3)	CT02—C2—Ba1—N2	−73.73 (16)
C6—C7—C8—CT01	0.30 (18)	C1—C2—Ba1—N2	−18.5 (2)
C17—C7—C8—CT01	−178.0 (4)	C3—C2—Ba1—N2	−130.12 (19)
Ba1—C7—C8—CT01	−71.40 (11)	C12—C2—Ba1—N2	106.4 (3)
CT01—C7—C8—C9	0.31 (19)	CT02—C2—Ba1—N1	−128.34 (15)
C6—C7—C8—C9	0.6 (3)	C1—C2—Ba1—N1	−73.06 (18)
C17—C7—C8—C9	−177.7 (3)	C3—C2—Ba1—N1	175.3 (2)
Ba1—C7—C8—C9	−71.1 (2)	C12—C2—Ba1—N1	51.8 (3)
CT01—C7—C8—C18	−176.3 (3)	CT02—C2—Ba1—C3	56.39 (18)
C6—C7—C8—C18	−176.0 (3)	C1—C2—Ba1—C3	111.7 (3)
C17—C7—C8—C18	5.7 (5)	C12—C2—Ba1—C3	−123.5 (4)
Ba1—C7—C8—C18	112.3 (3)	CT02—C2—Ba1—C7	118.30 (15)
CT01—C7—C8—Ba1	71.40 (11)	C1—C2—Ba1—C7	173.57 (17)
C6—C7—C8—Ba1	71.7 (2)	C3—C2—Ba1—C7	61.9 (2)
C17—C7—C8—Ba1	−106.6 (3)	C12—C2—Ba1—C7	−61.6 (3)
C7—C8—C9—CT01	−0.31 (19)	CT02—C2—Ba1—C8	85.13 (17)
C18—C8—C9—CT01	176.4 (3)	C1—C2—Ba1—C8	140.40 (17)
Ba1—C8—C9—CT01	−69.94 (11)	C3—C2—Ba1—C8	28.7 (2)
CT01—C8—C9—C10	−0.19 (18)	C12—C2—Ba1—C8	−94.8 (3)
C7—C8—C9—C10	−0.5 (3)	CT02—C2—Ba1—C6	141.68 (14)
C18—C8—C9—C10	176.2 (3)	C1—C2—Ba1—C6	−163.05 (17)
Ba1—C8—C9—C10	−70.1 (2)	C3—C2—Ba1—C6	85.3 (2)
CT01—C8—C9—C19	−178.5 (4)	C12—C2—Ba1—C6	−38.2 (3)
C7—C8—C9—C19	−178.8 (3)	CT02—C2—Ba1—C9	59.4 (2)
C18—C8—C9—C19	−2.1 (5)	C1—C2—Ba1—C9	114.6 (2)
Ba1—C8—C9—C19	111.6 (3)	C3—C2—Ba1—C9	3.0 (3)
CT01—C8—C9—Ba1	69.94 (11)	C12—C2—Ba1—C9	−120.5 (3)
C7—C8—C9—Ba1	69.6 (2)	CT02—C2—Ba1—C1	−55.27 (19)
C18—C8—C9—Ba1	−113.6 (3)	C3—C2—Ba1—C1	−111.7 (3)
C7—C6—C10—CT01	0.18 (18)	C12—C2—Ba1—C1	124.8 (3)
C16—C6—C10—CT01	−179.3 (3)	CT02—C2—Ba1—C10	89.3 (4)
Ba1—C6—C10—CT01	69.66 (11)	C1—C2—Ba1—C10	144.6 (4)
CT01—C6—C10—C9	0.00 (19)	C3—C2—Ba1—C10	32.9 (5)
C7—C6—C10—C9	0.2 (3)	C12—C2—Ba1—C10	−90.6 (4)
C16—C6—C10—C9	−179.3 (3)	CT02—C3—Ba1—CT01	151.48 (11)
Ba1—C6—C10—C9	69.7 (2)	C4—C3—Ba1—CT01	96.31 (19)
CT01—C6—C10—C20	178.3 (3)	C2—C3—Ba1—CT01	−152.16 (17)
C7—C6—C10—C20	178.5 (3)	C13—C3—Ba1—CT01	−28.8 (3)
C16—C6—C10—C20	−1.0 (5)	C4—C3—Ba1—CT02	−55.17 (19)
Ba1—C6—C10—C20	−112.0 (3)	C2—C3—Ba1—CT02	56.36 (19)
CT01—C6—C10—Ba1	−69.66 (11)	C13—C3—Ba1—CT02	179.7 (4)
C7—C6—C10—Ba1	−69.5 (2)	CT02—C3—Ba1—N2	5.2 (2)
C16—C6—C10—Ba1	111.1 (3)	C4—C3—Ba1—N2	−50.0 (2)
C8—C9—C10—CT01	0.19 (19)	C2—C3—Ba1—N2	61.5 (2)
C19—C9—C10—CT01	178.5 (4)	C13—C3—Ba1—N2	−175.1 (2)
Ba1—C9—C10—CT01	−69.10 (11)	CT02—C3—Ba1—N1	−61.69 (17)
CT01—C9—C10—C6	0.00 (19)	C4—C3—Ba1—N1	−116.86 (18)
C8—C9—C10—C6	0.2 (3)	C2—C3—Ba1—N1	−5.3 (2)
C19—C9—C10—C6	178.5 (3)	C13—C3—Ba1—N1	118.0 (3)
Ba1—C9—C10—C6	−69.1 (2)	CT02—C3—Ba1—C2	−56.36 (19)
CT01—C9—C10—C20	−178.3 (3)	C4—C3—Ba1—C2	−111.5 (3)
C8—C9—C10—C20	−178.1 (3)	C13—C3—Ba1—C2	123.3 (4)
C19—C9—C10—C20	0.2 (5)	CT02—C3—Ba1—C7	174.08 (14)
Ba1—C9—C10—C20	112.6 (3)	C4—C3—Ba1—C7	118.91 (19)
CT01—C9—C10—Ba1	69.10 (11)	C2—C3—Ba1—C7	−129.56 (19)
C8—C9—C10—Ba1	69.3 (2)	C13—C3—Ba1—C7	−6.2 (3)
C19—C9—C10—Ba1	−112.4 (3)	CT02—C3—Ba1—C8	147.88 (15)
N1—C21—C22—C23	1.5 (5)	C4—C3—Ba1—C8	92.71 (19)
C21—C22—C23—C24	−1.0 (5)	C2—C3—Ba1—C8	−155.76 (19)
C22—C23—C24—C25	0.0 (5)	C13—C3—Ba1—C8	−32.4 (3)
C23—C24—C25—N1	0.8 (5)	CT02—C3—Ba1—C6	175.60 (11)
C23—C24—C25—C26	−178.4 (3)	C4—C3—Ba1—C6	120.43 (19)
N1—C25—C26—N2	−1.7 (4)	C2—C3—Ba1—C6	−128.04 (19)
C24—C25—C26—N2	177.5 (3)	C13—C3—Ba1—C6	−4.7 (3)
N1—C25—C26—C27	179.7 (3)	CT02—C3—Ba1—C9	125.65 (14)
C24—C25—C26—C27	−1.1 (4)	C4—C3—Ba1—C9	70.5 (2)
N2—C26—C27—C28	0.2 (5)	C2—C3—Ba1—C9	−177.99 (18)
C25—C26—C27—C28	178.7 (3)	C13—C3—Ba1—C9	−54.7 (3)
C26—C27—C28—C29	−0.5 (5)	CT02—C3—Ba1—C1	−19.15 (12)
C27—C28—C29—C30	0.4 (6)	C4—C3—Ba1—C1	−74.3 (2)
C28—C29—C30—N2	0.0 (6)	C2—C3—Ba1—C1	37.21 (18)
C6—C10—CT01—C7	−0.3 (4)	C13—C3—Ba1—C1	160.5 (3)
C9—C10—CT01—C7	179.6 (3)	CT02—C3—Ba1—C10	134.67 (14)
C20—C10—CT01—C7	−99 (13)	C4—C3—Ba1—C10	79.5 (2)
Ba1—C10—CT01—C7	89.0 (3)	C2—C3—Ba1—C10	−168.97 (18)
C6—C10—CT01—C8	179.6 (3)	C13—C3—Ba1—C10	−45.7 (3)
C9—C10—CT01—C8	−0.4 (4)	C8—C7—Ba1—CT01	56.06 (16)
C20—C10—CT01—C8	81 (13)	C6—C7—Ba1—CT01	−56.19 (16)
Ba1—C10—CT01—C8	−91.0 (3)	C17—C7—Ba1—CT01	−179.5 (3)
C6—C10—CT01—C9	180.0 (2)	CT01—C7—Ba1—CT02	−122.84 (11)
C20—C10—CT01—C9	81 (13)	C8—C7—Ba1—CT02	−66.78 (18)
Ba1—C10—CT01—C9	−90.61 (15)	C6—C7—Ba1—CT02	−179.03 (13)
C9—C10—CT01—C6	180.0 (2)	C17—C7—Ba1—CT02	57.6 (2)
C20—C10—CT01—C6	−99 (13)	CT01—C7—Ba1—N2	42.46 (17)
Ba1—C10—CT01—C6	89.38 (15)	C8—C7—Ba1—N2	98.52 (18)
C6—C10—CT01—Ba1	−89.38 (15)	C6—C7—Ba1—N2	−13.7 (2)
C9—C10—CT01—Ba1	90.61 (15)	C17—C7—Ba1—N2	−137.1 (2)
C20—C10—CT01—Ba1	172 (100)	CT01—C7—Ba1—N1	105.04 (13)
C8—C7—CT01—C10	180.0 (3)	C8—C7—Ba1—N1	161.10 (16)
C6—C7—CT01—C10	0.3 (4)	C6—C7—Ba1—N1	48.85 (18)
C17—C7—CT01—C10	−75 (11)	C17—C7—Ba1—N1	−74.5 (2)
Ba1—C7—CT01—C10	−90.0 (3)	CT01—C7—Ba1—C2	−151.85 (12)
C6—C7—CT01—C8	−179.6 (2)	C8—C7—Ba1—C2	−95.79 (18)
C17—C7—CT01—C8	105 (11)	C6—C7—Ba1—C2	151.96 (16)
Ba1—C7—CT01—C8	90.05 (15)	C17—C7—Ba1—C2	28.6 (2)
C8—C7—CT01—C9	−0.6 (4)	CT01—C7—Ba1—C3	−126.10 (13)
C6—C7—CT01—C9	179.8 (3)	C8—C7—Ba1—C3	−70.04 (18)
C17—C7—CT01—C9	105 (10)	C6—C7—Ba1—C3	177.71 (17)
Ba1—C7—CT01—C9	89.5 (3)	C17—C7—Ba1—C3	54.4 (2)
C8—C7—CT01—C6	179.6 (2)	CT01—C7—Ba1—C8	−56.06 (16)
C17—C7—CT01—C6	−75 (10)	C6—C7—Ba1—C8	−112.3 (2)
Ba1—C7—CT01—C6	−90.31 (15)	C17—C7—Ba1—C8	124.4 (3)
C8—C7—CT01—Ba1	−90.05 (15)	CT01—C7—Ba1—C6	56.19 (16)
C6—C7—CT01—Ba1	90.31 (15)	C8—C7—Ba1—C6	112.3 (2)
C17—C7—CT01—Ba1	15 (10)	C17—C7—Ba1—C6	−123.3 (3)
C9—C8—CT01—C10	0.4 (4)	CT01—C7—Ba1—C9	−19.48 (11)
C7—C8—CT01—C10	−180.0 (3)	C8—C7—Ba1—C9	36.58 (16)
C18—C8—CT01—C10	−76 (6)	C6—C7—Ba1—C9	−75.67 (18)
Ba1—C8—CT01—C10	91.5 (3)	C17—C7—Ba1—C9	161.0 (3)
C9—C8—CT01—C7	−179.6 (2)	CT01—C7—Ba1—C1	−145.72 (15)
C18—C8—CT01—C7	104 (6)	C8—C7—Ba1—C1	−89.7 (2)
Ba1—C8—CT01—C7	−88.48 (15)	C6—C7—Ba1—C1	158.10 (18)
C7—C8—CT01—C9	179.6 (2)	C17—C7—Ba1—C1	34.8 (3)
C18—C8—CT01—C9	−77 (6)	CT01—C7—Ba1—C10	19.40 (11)
Ba1—C8—CT01—C9	91.16 (15)	C8—C7—Ba1—C10	75.46 (18)
C9—C8—CT01—C6	179.8 (3)	C6—C7—Ba1—C10	−36.79 (16)
C7—C8—CT01—C6	−0.6 (4)	C17—C7—Ba1—C10	−160.1 (3)
C18—C8—CT01—C6	103 (6)	C9—C8—Ba1—CT01	56.19 (16)
Ba1—C8—CT01—C6	−89.1 (3)	C7—C8—Ba1—CT01	−56.65 (16)
C9—C8—CT01—Ba1	−91.16 (15)	C18—C8—Ba1—CT01	179.3 (3)
C7—C8—CT01—Ba1	88.48 (15)	CT01—C8—Ba1—CT02	−173.48 (10)
C18—C8—CT01—Ba1	−168 (6)	C9—C8—Ba1—CT02	−117.30 (15)
C8—C9—CT01—C10	−179.8 (2)	C7—C8—Ba1—CT02	129.87 (15)
C19—C9—CT01—C10	−77 (13)	C18—C8—Ba1—CT02	5.8 (2)
Ba1—C9—CT01—C10	91.00 (15)	CT01—C8—Ba1—N2	−45.74 (18)
C8—C9—CT01—C7	0.6 (4)	C9—C8—Ba1—N2	10.4 (2)
C10—C9—CT01—C7	−179.6 (3)	C7—C8—Ba1—N2	−102.39 (18)
C19—C9—CT01—C7	103 (13)	C18—C8—Ba1—N2	133.5 (2)
Ba1—C9—CT01—C7	−88.6 (3)	CT01—C8—Ba1—N1	30.91 (19)
C10—C9—CT01—C8	179.8 (2)	C9—C8—Ba1—N1	87.09 (19)
C19—C9—CT01—C8	103 (13)	C7—C8—Ba1—N1	−25.7 (2)
Ba1—C9—CT01—C8	−89.22 (15)	C18—C8—Ba1—N1	−149.8 (2)
C8—C9—CT01—C6	−179.8 (3)	CT01—C8—Ba1—C2	159.24 (12)
C10—C9—CT01—C6	0.0 (4)	C9—C8—Ba1—C2	−144.58 (16)
C19—C9—CT01—C6	−77 (13)	C7—C8—Ba1—C2	102.59 (17)
Ba1—C9—CT01—C6	91.0 (3)	C18—C8—Ba1—C2	−21.5 (3)
C8—C9—CT01—Ba1	89.22 (15)	CT01—C8—Ba1—C3	172.35 (14)
C10—C9—CT01—Ba1	−91.00 (15)	C9—C8—Ba1—C3	−131.46 (17)
C19—C9—CT01—Ba1	−168 (100)	C7—C8—Ba1—C3	115.70 (17)
C7—C6—CT01—C10	−179.8 (2)	C18—C8—Ba1—C3	−8.4 (2)
C16—C6—CT01—C10	53 (24)	CT01—C8—Ba1—C7	56.65 (16)
Ba1—C6—CT01—C10	−91.38 (15)	C9—C8—Ba1—C7	112.8 (2)
C10—C6—CT01—C7	179.8 (2)	C18—C8—Ba1—C7	−124.1 (3)
C16—C6—CT01—C7	−128 (24)	CT01—C8—Ba1—C6	19.45 (11)
Ba1—C6—CT01—C7	88.40 (15)	C9—C8—Ba1—C6	75.64 (18)
C10—C6—CT01—C8	−179.6 (3)	C7—C8—Ba1—C6	−37.20 (16)
C7—C6—CT01—C8	0.6 (4)	C18—C8—Ba1—C6	−161.3 (3)
C16—C6—CT01—C8	−127 (24)	CT01—C8—Ba1—C9	−56.19 (16)
Ba1—C6—CT01—C8	89.0 (3)	C7—C8—Ba1—C9	−112.8 (2)
C10—C6—CT01—C9	0.0 (4)	C18—C8—Ba1—C9	123.1 (3)
C7—C6—CT01—C9	−179.8 (3)	CT01—C8—Ba1—C1	−173.09 (12)
C16—C6—CT01—C9	53 (24)	C9—C8—Ba1—C1	−116.91 (19)
Ba1—C6—CT01—C9	−91.4 (3)	C7—C8—Ba1—C1	130.26 (18)
C10—C6—CT01—Ba1	91.38 (15)	C18—C8—Ba1—C1	6.2 (3)
C7—C6—CT01—Ba1	−88.40 (15)	CT01—C8—Ba1—C10	−19.45 (11)
C16—C6—CT01—Ba1	144 (24)	C9—C8—Ba1—C10	36.74 (16)
C3—C4—CT02—C1	179.7 (3)	C7—C8—Ba1—C10	−76.10 (18)
C5—C4—CT02—C1	−0.1 (4)	C18—C8—Ba1—C10	159.8 (3)
C14—C4—CT02—C1	59 (14)	C10—C6—Ba1—CT01	−55.78 (16)
Ba1—C4—CT02—C1	−93.1 (3)	C7—C6—Ba1—CT01	56.60 (17)
C3—C4—CT02—C2	−0.5 (4)	C16—C6—Ba1—CT01	−179.5 (3)
C5—C4—CT02—C2	179.7 (3)	CT01—C6—Ba1—CT02	−54.5 (3)
C14—C4—CT02—C2	−121 (14)	C10—C6—Ba1—CT02	−110.30 (19)
Ba1—C4—CT02—C2	86.7 (3)	C7—C6—Ba1—CT02	2.1 (3)
C3—C4—CT02—C5	179.9 (2)	C16—C6—Ba1—CT02	125.9 (2)
C14—C4—CT02—C5	59 (14)	CT01—C6—Ba1—N2	112.28 (14)
Ba1—C4—CT02—C5	−92.97 (16)	C10—C6—Ba1—N2	56.50 (17)
C5—C4—CT02—C3	−179.9 (2)	C7—C6—Ba1—N2	168.88 (17)
C14—C4—CT02—C3	−121 (14)	C16—C6—Ba1—N2	−67.3 (2)
Ba1—C4—CT02—C3	87.14 (16)	CT01—C6—Ba1—N1	168.61 (14)
C3—C4—CT02—Ba1	−87.14 (16)	C10—C6—Ba1—N1	112.83 (16)
C5—C4—CT02—Ba1	92.97 (16)	C7—C6—Ba1—N1	−134.78 (17)
C14—C4—CT02—Ba1	152 (14)	C16—C6—Ba1—N1	−10.9 (2)
C5—C1—CT02—C4	0.1 (4)	CT01—C6—Ba1—C2	−101.64 (18)
C2—C1—CT02—C4	179.9 (3)	C10—C6—Ba1—C2	−157.42 (16)
C11—C1—CT02—C4	−95 (34)	C7—C6—Ba1—C2	−45.0 (2)
Ba1—C1—CT02—C4	93.2 (3)	C16—C6—Ba1—C2	78.8 (3)
C5—C1—CT02—C2	−179.7 (2)	CT01—C6—Ba1—C3	−59.72 (19)
C11—C1—CT02—C2	85 (34)	C10—C6—Ba1—C3	−115.50 (17)
Ba1—C1—CT02—C2	−86.63 (16)	C7—C6—Ba1—C3	−3.1 (2)
C2—C1—CT02—C5	179.7 (2)	C16—C6—Ba1—C3	120.7 (2)
C11—C1—CT02—C5	−95 (34)	CT01—C6—Ba1—C7	−56.60 (17)
Ba1—C1—CT02—C5	93.10 (16)	C10—C6—Ba1—C7	−112.4 (2)
C5—C1—CT02—C3	179.7 (3)	C16—C6—Ba1—C7	123.9 (3)
C2—C1—CT02—C3	−0.5 (4)	CT01—C6—Ba1—C8	−19.41 (11)
C11—C1—CT02—C3	84 (34)	C10—C6—Ba1—C8	−75.19 (17)
Ba1—C1—CT02—C3	−87.2 (3)	C7—C6—Ba1—C8	37.19 (16)
C5—C1—CT02—Ba1	−93.10 (16)	C16—C6—Ba1—C8	161.1 (3)
C2—C1—CT02—Ba1	86.63 (16)	CT01—C6—Ba1—C9	19.46 (11)
C11—C1—CT02—Ba1	172 (100)	C10—C6—Ba1—C9	−36.32 (15)
C1—C2—CT02—C4	−179.9 (3)	C7—C6—Ba1—C9	76.06 (18)
C3—C2—CT02—C4	0.5 (4)	C16—C6—Ba1—C9	−160.1 (3)
C12—C2—CT02—C4	−87 (6)	CT01—C6—Ba1—C1	−149.8 (4)
Ba1—C2—CT02—C4	−88.1 (3)	C10—C6—Ba1—C1	154.4 (4)
C3—C2—CT02—C1	−179.7 (2)	C7—C6—Ba1—C1	−93.2 (5)
C12—C2—CT02—C1	93 (6)	C16—C6—Ba1—C1	30.7 (6)
Ba1—C2—CT02—C1	91.82 (16)	CT01—C6—Ba1—C10	55.78 (16)
C1—C2—CT02—C5	−0.4 (4)	C7—C6—Ba1—C10	112.4 (2)
C3—C2—CT02—C5	179.9 (3)	C16—C6—Ba1—C10	−123.7 (3)
C12—C2—CT02—C5	93 (6)	C8—C9—Ba1—CT01	−56.89 (16)
Ba1—C2—CT02—C5	91.4 (3)	C10—C9—Ba1—CT01	56.00 (16)
C1—C2—CT02—C3	179.7 (2)	C19—C9—Ba1—CT01	179.7 (3)
C12—C2—CT02—C3	−87 (6)	CT01—C9—Ba1—CT02	135.92 (10)
Ba1—C2—CT02—C3	−88.51 (16)	C8—C9—Ba1—CT02	79.03 (17)
C1—C2—CT02—Ba1	−91.82 (15)	C10—C9—Ba1—CT02	−168.08 (14)
C3—C2—CT02—Ba1	88.51 (16)	C19—C9—Ba1—CT02	−44.4 (2)
C12—C2—CT02—Ba1	2 (6)	CT01—C9—Ba1—N2	−114.55 (13)
C1—C5—CT02—C4	−179.9 (2)	C8—C9—Ba1—N2	−171.44 (16)
C15—C5—CT02—C4	−99 (7)	C10—C9—Ba1—N2	−58.55 (18)
Ba1—C5—CT02—C4	90.28 (16)	C19—C9—Ba1—N2	65.2 (2)
C4—C5—CT02—C1	179.9 (2)	CT01—C9—Ba1—N1	−60.22 (16)
C15—C5—CT02—C1	81 (7)	C8—C9—Ba1—N1	−117.11 (17)
Ba1—C5—CT02—C1	−89.81 (16)	C10—C9—Ba1—N1	−4.2 (2)
C1—C5—CT02—C2	0.4 (4)	C19—C9—Ba1—N1	119.5 (2)
C4—C5—CT02—C2	−179.7 (3)	CT01—C9—Ba1—C2	109.99 (16)
C15—C5—CT02—C2	82 (7)	C8—C9—Ba1—C2	53.1 (2)
Ba1—C5—CT02—C2	−89.4 (3)	C10—C9—Ba1—C2	165.99 (17)
C1—C5—CT02—C3	−179.7 (3)	C19—C9—Ba1—C2	−70.3 (3)
C4—C5—CT02—C3	0.2 (4)	CT01—C9—Ba1—C3	111.52 (14)
C15—C5—CT02—C3	−99 (7)	C8—C9—Ba1—C3	54.63 (19)
Ba1—C5—CT02—C3	90.5 (3)	C10—C9—Ba1—C3	167.52 (17)
C1—C5—CT02—Ba1	89.81 (16)	C19—C9—Ba1—C3	−68.8 (2)
C4—C5—CT02—Ba1	−90.28 (16)	CT01—C9—Ba1—C7	19.78 (11)
C15—C5—CT02—Ba1	171 (7)	C8—C9—Ba1—C7	−37.10 (16)
C2—C3—CT02—C4	−179.7 (2)	C10—C9—Ba1—C7	75.79 (18)
C13—C3—CT02—C4	97 (11)	C19—C9—Ba1—C7	−160.5 (3)
Ba1—C3—CT02—C4	−92.07 (16)	CT01—C9—Ba1—C8	56.89 (16)
C4—C3—CT02—C1	−179.7 (3)	C10—C9—Ba1—C8	112.9 (2)
C2—C3—CT02—C1	0.5 (4)	C19—C9—Ba1—C8	−123.4 (3)
C13—C3—CT02—C1	−83 (11)	CT01—C9—Ba1—C6	−19.66 (11)
Ba1—C3—CT02—C1	88.2 (3)	C8—C9—Ba1—C6	−76.55 (18)
C4—C3—CT02—C2	179.7 (2)	C10—C9—Ba1—C6	36.34 (16)
C13—C3—CT02—C2	−84 (11)	C19—C9—Ba1—C6	160.0 (3)
Ba1—C3—CT02—C2	87.65 (16)	CT01—C9—Ba1—C1	156.97 (13)
C4—C3—CT02—C5	−0.2 (4)	C8—C9—Ba1—C1	100.1 (2)
C2—C3—CT02—C5	−179.9 (3)	C10—C9—Ba1—C1	−147.03 (17)
C13—C3—CT02—C5	96 (11)	C19—C9—Ba1—C1	−23.3 (3)
Ba1—C3—CT02—C5	−92.3 (3)	CT01—C9—Ba1—C10	−56.00 (16)
C4—C3—CT02—Ba1	92.07 (16)	C8—C9—Ba1—C10	−112.9 (2)
C2—C3—CT02—Ba1	−87.65 (16)	C19—C9—Ba1—C10	123.7 (3)
C13—C3—CT02—Ba1	−171 (100)	CT02—C1—Ba1—CT01	−11.2 (4)
C22—C21—N1—C25	−0.8 (5)	C5—C1—Ba1—CT01	44.7 (4)
C22—C21—N1—Ba1	167.2 (3)	C2—C1—Ba1—CT01	−68.6 (3)
C24—C25—N1—C21	−0.4 (4)	C11—C1—Ba1—CT01	168.9 (2)
C26—C25—N1—C21	178.9 (3)	C5—C1—Ba1—CT02	55.89 (18)
C24—C25—N1—Ba1	−168.0 (2)	C2—C1—Ba1—CT02	−57.43 (18)
C26—C25—N1—Ba1	11.2 (3)	C11—C1—Ba1—CT02	−179.9 (4)
C29—C30—N2—C26	−0.3 (5)	CT02—C1—Ba1—N2	−140.24 (17)
C29—C30—N2—Ba1	−170.6 (3)	C5—C1—Ba1—N2	−84.4 (2)
C27—C26—N2—C30	0.2 (4)	C2—C1—Ba1—N2	162.32 (19)
C25—C26—N2—C30	−178.4 (3)	C11—C1—Ba1—N2	39.9 (3)
C27—C26—N2—Ba1	169.3 (2)	CT02—C1—Ba1—N1	162.34 (17)
C25—C26—N2—Ba1	−9.3 (4)	C5—C1—Ba1—N1	−141.8 (2)
C10—CT01—Ba1—CT02	−134.72 (14)	C2—C1—Ba1—N1	104.91 (19)
C7—CT01—Ba1—CT02	81.37 (14)	C11—C1—Ba1—N1	−17.5 (3)
C8—CT01—Ba1—CT02	9.20 (14)	CT02—C1—Ba1—C2	57.43 (18)
C9—CT01—Ba1—CT02	−62.52 (14)	C5—C1—Ba1—C2	113.3 (3)
C6—CT01—Ba1—CT02	153.47 (14)	C11—C1—Ba1—C2	−122.4 (4)
C10—CT01—Ba1—N2	−1.50 (15)	CT02—C1—Ba1—C3	19.66 (13)
C7—CT01—Ba1—N2	−145.41 (15)	C5—C1—Ba1—C3	75.6 (2)
C8—CT01—Ba1—N2	142.42 (15)	C2—C1—Ba1—C3	−37.77 (17)
C9—CT01—Ba1—N2	70.70 (15)	C11—C1—Ba1—C3	−160.2 (3)
C6—CT01—Ba1—N2	−73.31 (15)	CT02—C1—Ba1—C7	46.4 (3)
C10—CT01—Ba1—N1	59.34 (15)	C5—C1—Ba1—C7	102.2 (2)
C7—CT01—Ba1—N1	−84.57 (15)	C2—C1—Ba1—C7	−11.1 (3)
C8—CT01—Ba1—N1	−156.74 (15)	C11—C1—Ba1—C7	−133.5 (3)
C9—CT01—Ba1—N1	131.54 (15)	CT02—C1—Ba1—C8	−0.9 (3)
C6—CT01—Ba1—N1	−12.47 (15)	C5—C1—Ba1—C8	55.0 (3)
C10—CT01—Ba1—C2	−174.49 (17)	C2—C1—Ba1—C8	−58.3 (2)
C7—CT01—Ba1—C2	41.60 (17)	C11—C1—Ba1—C8	179.2 (2)
C8—CT01—Ba1—C2	−30.57 (18)	CT02—C1—Ba1—C6	120.2 (4)
C9—CT01—Ba1—C2	−102.29 (17)	C5—C1—Ba1—C6	176.1 (4)
C6—CT01—Ba1—C2	113.70 (18)	C2—C1—Ba1—C6	62.8 (5)
C10—CT01—Ba1—C3	−152.75 (16)	C11—C1—Ba1—C6	−59.7 (6)
C7—CT01—Ba1—C3	63.34 (16)	CT02—C1—Ba1—C9	−46.4 (3)
C8—CT01—Ba1—C3	−8.83 (16)	C5—C1—Ba1—C9	9.4 (3)
C9—CT01—Ba1—C3	−80.55 (16)	C2—C1—Ba1—C9	−103.9 (2)
C6—CT01—Ba1—C3	135.44 (16)	C11—C1—Ba1—C9	133.7 (3)
C10—CT01—Ba1—C7	143.91 (19)	CT02—C1—Ba1—C10	−98.5 (3)
C8—CT01—Ba1—C7	−72.17 (19)	C5—C1—Ba1—C10	−42.6 (4)
C9—CT01—Ba1—C7	−143.89 (19)	C2—C1—Ba1—C10	−156.0 (2)
C6—CT01—Ba1—C7	72.1 (2)	C11—C1—Ba1—C10	81.6 (4)
C10—CT01—Ba1—C8	−143.9 (2)	C6—C10—Ba1—CT01	57.19 (17)
C7—CT01—Ba1—C8	72.17 (19)	C9—C10—Ba1—CT01	−56.53 (16)
C9—CT01—Ba1—C8	−71.72 (19)	C20—C10—Ba1—CT01	−179.8 (3)
C6—CT01—Ba1—C8	144.3 (2)	CT01—C10—Ba1—CT02	77.78 (19)
C10—CT01—Ba1—C6	71.8 (2)	C6—C10—Ba1—CT02	134.97 (15)
C7—CT01—Ba1—C6	−72.1 (2)	C9—C10—Ba1—CT02	21.3 (2)
C8—CT01—Ba1—C6	−144.3 (2)	C20—C10—Ba1—CT02	−102.0 (2)
C9—CT01—Ba1—C6	144.01 (19)	CT01—C10—Ba1—N2	178.56 (15)
C10—CT01—Ba1—C9	−72.20 (19)	C6—C10—Ba1—N2	−124.25 (17)
C7—CT01—Ba1—C9	143.89 (19)	C9—C10—Ba1—N2	122.03 (18)
C8—CT01—Ba1—C9	71.72 (19)	C20—C10—Ba1—N2	−1.2 (2)
C6—CT01—Ba1—C9	−144.01 (19)	CT01—C10—Ba1—N1	−126.85 (13)
C10—CT01—Ba1—C1	−127.7 (3)	C6—C10—Ba1—N1	−69.66 (16)
C7—CT01—Ba1—C1	88.4 (3)	C9—C10—Ba1—N1	176.62 (16)
C8—CT01—Ba1—C1	16.2 (3)	C20—C10—Ba1—N1	53.4 (2)
C9—CT01—Ba1—C1	−55.5 (3)	CT01—C10—Ba1—C2	14.7 (5)
C6—CT01—Ba1—C1	160.5 (3)	C6—C10—Ba1—C2	71.9 (4)
C7—CT01—Ba1—C10	−143.9 (2)	C9—C10—Ba1—C2	−41.8 (5)
C8—CT01—Ba1—C10	143.9 (2)	C20—C10—Ba1—C2	−165.0 (3)
C9—CT01—Ba1—C10	72.20 (19)	CT01—C10—Ba1—C3	38.4 (2)
C6—CT01—Ba1—C10	−71.8 (2)	C6—C10—Ba1—C3	95.6 (2)
C4—CT02—Ba1—CT01	31.32 (17)	C9—C10—Ba1—C3	−18.1 (2)
C1—CT02—Ba1—CT01	175.20 (17)	C20—C10—Ba1—C3	−141.4 (2)
C2—CT02—Ba1—CT01	−112.71 (16)	CT01—C10—Ba1—C7	−19.83 (11)
C5—CT02—Ba1—CT01	103.52 (16)	C6—C10—Ba1—C7	37.36 (16)
C3—CT02—Ba1—CT01	−40.52 (16)	C9—C10—Ba1—C7	−76.36 (18)
C4—CT02—Ba1—N2	−103.82 (18)	C20—C10—Ba1—C7	160.4 (3)
C1—CT02—Ba1—N2	40.05 (18)	CT01—C10—Ba1—C8	19.74 (11)
C2—CT02—Ba1—N2	112.15 (17)	C6—C10—Ba1—C8	76.93 (17)
C5—CT02—Ba1—N2	−31.62 (17)	C9—C10—Ba1—C8	−36.79 (16)
C3—CT02—Ba1—N2	−175.66 (16)	C20—C10—Ba1—C8	−160.0 (3)
C4—CT02—Ba1—N1	−161.93 (18)	CT01—C10—Ba1—C6	−57.19 (17)
C1—CT02—Ba1—N1	−18.05 (18)	C9—C10—Ba1—C6	−113.7 (2)
C2—CT02—Ba1—N1	54.04 (17)	C20—C10—Ba1—C6	123.0 (3)
C5—CT02—Ba1—N1	−89.73 (17)	CT01—C10—Ba1—C9	56.53 (16)
C3—CT02—Ba1—N1	126.23 (17)	C6—C10—Ba1—C9	113.7 (2)
C4—CT02—Ba1—C2	144.0 (2)	C20—C10—Ba1—C9	−123.2 (3)
C1—CT02—Ba1—C2	−72.1 (2)	CT01—C10—Ba1—C1	137.1 (2)
C5—CT02—Ba1—C2	−143.8 (2)	C6—C10—Ba1—C1	−165.7 (2)
C3—CT02—Ba1—C2	72.2 (2)	C9—C10—Ba1—C1	80.5 (3)
C4—CT02—Ba1—C3	71.8 (2)	C20—C10—Ba1—C1	−42.7 (4)
